# Bacteremia Caused by Group G Streptococci, Taiwan

**DOI:** 10.3201/eid1405.070130

**Published:** 2008-05

**Authors:** Chun-Hsing Liao, Liang-Chun Liu, Yu-Tsung Huang, Lee-Jeng Teng, Po-Ren Hsueh

**Affiliations:** * Far-Eastern Memorial Hospital, Taipei, Taiwan; † National Taiwan University College of Medicine, Taipei, Taiwan

**Keywords:** Group G streptococcus, bacteremia, Taiwan, dispatch

## Abstract

A retrospective observational study in Taiwan, 1998–2004, identified 92 patients with group G streptococcal bacteremia; 86 had *Streptococcus dysgalactiae* subspecies *equisimilis*. The most common diagnosis was cellulitis (48 cases), followed by primary bacteremia (34 cases). Infection recurred in 9 patients. Mortality rate was low (3.3%); resistance to quinupristin-dalfopristin was high.

Group G streptococci (GGS) are part of the normal microbial flora of the gastrointestinal tract, vagina, and skin and cause a variety of infections (*1*). Major underlying illnesses in patients with GGS bacteremia are malignancy, cardiovascular disease, diabetes mellitus, bone and joint diseases, and cirrhosis (*1**,**2*). Reported mortality rates for patients with GGS bacteremia also vary, ranging from 5% to 30% (*1**–**3*). Recent studies of β-hemolytic streptococci isolates carrying Lancefield group G antigen showed that they consist of *Streptococcus dysgalactiae* subspecies *equisimilis*, *S. anginosus*, and *S. canis* (*2**,**4**–**6*). To supplement the limited clinical information about bacteremia caused by GGS strains identified to the species level (*2**–**4*), we conducted a retrospective observational study.

## The Study

We included all patients with GGS-positive blood cultures who had been treated from April 1998 through August 2004 at National Taiwan University Hospital, a 2,000-bed teaching hospital in northern Taiwan. We recorded demographic parameters, underlying illness, clinical diagnosis, and outcome for each patient. Clinical diagnosis was based on the attending physician’s judgment and examination results. Recurrence of bacteremia was defined as repeated positive blood culture after complete treatment (at least 14 days) of previous bacteremia.

Differentiation of GGS was based on colony size, hemolytic reaction, Voges-Proskauer reaction, and β-glucuronidase activity. All β-hemolytic streptococci, whether large or small colonies, were tested for Lancefield group by using an agglutination kit (Streptex; Murex Biotech Ltd., Dartford, UK). PCR to differentiate between *S. anginosus* and *S. dysgalactiae* subsp. *equisimilis* was performed for all GGS isolates as described (*7*). For identification of *S. canis*, a probable isolate was identified by a negative β-glucuronidase result and further confirmed with the 16sRNA method as described (*8*). Susceptibilities of these isolates were tested by using the broth microdilution method as defined by the Clinical and Laboratory Standards Institute (formerly National Committee for Clinical Laboratory Standards) (*9*).

To determine the similarity of isolates in cases of recurrence, we used pulsed-field gel electrophoresis (PFGE) as described (*10*). The *emm* typing of isolates in cases of recurrence were also determined as described (*11*). The first 160 bases sequenced by emmseq2 that had >95% identity were defined as having the same genotype (*11*).

During the study period, 106 episodes of GGS bacteremia in 92 patients had been recorded; 56 episodes occurred during the first half of the study period (before June 2001) and 50 episodes during the second half. The causative agent was *S. dysgalactiae* subsp. *equisimilis* for 99 episodes, *S. anginosus* for 5, and *S. canis* for 2. Bacteremia recurred for 9 patients (1 had 4 episodes, and 3 had 3 episodes); bacteremia was nosocomial for 7 patients and polymicrobial for 5. The clinical characteristics of the patients are summarized in Table 1. All 3 patients who died had a diagnosis of the primary bacteremia caused by S**.** *dysgalactiae* subsp. *equisimilis*.

**Table 1:** Clinical characteristic of 92 patients with group G streptococcal bacteremia, April 1998–August 2004, Taiwan

Characteristic	No. (%) patients
Age, y	
<10	1 (1.1)
10–50	12 (13.0)
51–75	68 (73.9)
>75	11 (12.0)
Median (range)	72 (10–93)
Sex	
Male	58 (63.0)
Female	34 (37.0)
Underlying diseases	
Malignancy	35 (38.0)
Genital	10 (10.9)
Head and neck	8 (8.7)
Gastrointestinal	6 (6.5)
Hematologic	3 (3.3)
Tissue edema	25 (27.2)
Heart disease	20 (21.7)
Post–coronary artery bypass graft	6 (6.5)
Diabetes mellitus	16 (17.4)
Central nervous system disease	15 (16.3)
Liver cirrhosis	9 (9.8)
Chronic renal disease	8 (8.7)
Chronic lung disease	6 (6.5)
Bone disease	5 (5.4)
Deep venous thrombosis	2 (2.2)
Type of infection	
Cellulitis	48 (52.1)*
Primary bacteremia	34 (36.9)
Deep-seated abscess	4 (4.2)†
Neutropenia and fever	3 (3.3)
Septic arthritis	2 (2.2)
Urinary tract infection	1 (1.1)
Infective endocarditis	1 (1.1)
Pneumonia	1 (1.1)
Initial findings	
Fever	86 (93.5)
Leukocytosis (>10,000 cells/μL)	34 (37.0)
Septic shock	4 (5.4)
Outcome	
Death	3 (3.3)
Recurrence of bacteremia	9 (9.8)

*Includes 2 patients who also had septic arthritis. †Includes 2 patients with psoas muscle abscess, 1 with epidural abscess, and 1 with deep neck infection.

Among the 9 patients with recurrent bacteremia, the causative agent was *S. dysgalactiae* subsp. *equisimilis* for 8 and *S. canis* for 1. PFGE performed with all 13 available isolates from recurrent cases showed that 10 were identical to that of the initial episode, including 1 in a patient with recurrence of *S. canis* bacteremia. Sequence typing showed *emm* type stG485 for 4 patients. The clinical characteristics of the patients and *emm* typing results are shown in Table 2; PFGE results are shown in the Figure. The underlying diseases of patients with recurrent episodes included genital cancer (4 [44.4%] patients) and history of cellulitis (6 [66.7%]), each of which was significantly correlated with the likelihood of recurrence (p<0.01 for each). Further analysis showed that a previous history of cellulitis was significantly correlated with female sex (p = 0.01), genital cancer (p<0.01), tissue edema (p = 0.02), heart disease (p = 0.04), and post–coronary artery bypass graft (p = 0.03).

**Table 2:** Summary of characteristics of patients with recurrence of group G streptococcus bacteremia, April 1998–August 2004, Taiwan*

Patient no.	Age, y/ sex	Isolate	Date of isolation	Underlying disease	Clinical diagnosis	*emm* type	PFGE pattern
1	67/F	A1	2001 May 28	Coronary heart disease, post–coronary artery bypass graft	Cellulitis	stG166b	–
		A2	2002 Jul 18		Cellulitis	stG166b	Identical
		A3	2003 Oct 14		Cellulitis	stG166b	Identical
2	33/M	B1†	2002 Nov 13	Alcoholic liver cirrhosis, child C	Primary bacteremia	STL1929.1	–
		B2†	2002 Oct 15		Primary bacteremia	STL1929.1	Identical
3	47/F	C1	1998 May 15	Vulvar cancer after surgery and radiotherapy	Cellulitis	stG166b	–
		C2	2002 Jan 18		Cellulitis	stG6.1	Related
		C3	2002 Dec 19		Cellulitis	stG6.1	Identical
4	49/M	D1	2000 May 24	Nasopharyngeal carcinoma after chemotherapy and radiotherapy	Cellulitis	stG485	–
		D2	2000 Aug 9		Cellulitis	stG485	Identical
5	28/M	E1	1998 Dec 26	von Willebrand disease, type I	Cellulitis	stG485	–
		E2	1999 Aug 28		Cellulitis	stG840	Different
6	72/F	F1	1998 Aug 24	Cervical cancer after surgery and radiotherapy, diabetes mellitus	Cellulitis	stG485	–
		F2	1998 Oct 23		Cellulitis	stG485	Identical
		F3	1999 Dec 3		Cellulitis	stG840	Different
7	55/F	G1	1999 Oct 9	Cervical cancer after surgery and radiotherapy	Cellulitis	stG485	–
		G2	2000 Apr 18		Cellulitis	stG485	Identical
		G3	2001 Sep 24		Cellulitis	stG485	Identical
		NA	2000 Jul 19		Cellulitis	NA	NA
8	46/M	H1	2001 Aug 21	Acute myeloid leukemia (M4)	Primary bacteremia	stGLP 1.0	–
		H2	2001 Sep 6		Primary bacteremia	stGLP 1.0	Identical
9	80/F	I1	2003 May 5	Cervical cancer with lung metastasis and obstructive uropathy	Primary bacteremia	stG245.0	–
		I2	2003 Nov 17		Primary bacteremia	stG245.0	Identical

*PFGE, pulsed-field gel electrophoresis; NA, not available. †*Streptoccus canis*.

**Figure:**
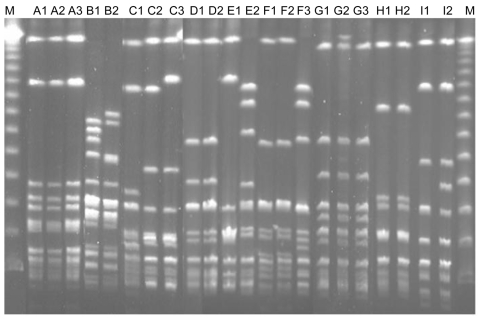
Pulsed-field gel electrophoresis profiles of all isolates from patients with recurrent group G streptococcal bacteremia. Isolates B1 and B2, *Streptococcus canis;* other isolates, *S. dysgalactiae* subsp. *equisimilis* (see designation of the isolates in Table 2). Lane M, molecular mass marker.

Bacteremia caused by β-hemolytic *S. anginosus* with group G antigen was identified for 5 patients, none of whom had cellulitis, compared with 48 (55.8%) of the 86 patients with *S. dysgalactiae* subsp. *equisimilis* who did have cellulitis (p = 0.03). Polymicrobial bacteremia and nosocomial bacteremia were found in a higher percentage of patients with *S. anginosus* (60% and 40.0%, respectively) than of patients with *S. dysgalactiae* subsp. *equisimilis* bacteremia (4.7% and 5.8%, respectively); p<0.01 and p = 0.02, respectively. The 1 patient with *S. canis* bacteremia was a 33-year-old man with no history of dog bite. He had alcohol-associated liver cirrhosis of Child C (severe) classification and leg edema. He had 2 episodes of *S. canis* bacteremia 1 month apart. Echocardiogram results showed no evidence of valvular vegetation. For the first episode, the patient received a 14-day course of cefotaxime.

Antimicrobial drug–susceptibility testing showed decreased susceptibility to only macrolides (susceptibity rates: azithromycin 67.4%, clarithromycin 73.9%), clindamycin (87.0%), and quinupristin-dalfopristin (33.7%) (Appendix Table). No clinical factor correlated with macrolide resistance. All isolates of recurrent bacteremia were susceptible to macrolides.

## Conclusions

We documented 5 cases of primary bacteremia caused by β-hemolytic group G *S. anginosus* and unintentionally documented recurrence of *S. canis* bacteremia. *S. canis* bacteremia in humans was first clearly described in 1997 (*12*).

Our finding of 5 β-hemolytic *S. anginosus* isolates and 1 *S. canis* isolate in patients with GGS bacteremia in this study differs from findings of previous studies (*2**,**3*). Factors that may have contributed to this discrepancy include serotype determination and PCR method. Serotype determination was performed for all β-hemolytic streptococci isolated in our hospital, whether colonies were large or small, which might have led to the detection of more streptococcal isolates with G antigen. The PCR method developed in our hospital and used in this study could effectively differentiate *S. anginosus* from *S. dysgalactiae* subsp. *equisimilis* (*7*).

Information about clinical infection with *S. milleri* with group G antigen is limited (*4*). In a previous study of GGS bacteremia, Cohen-Poradosu et al. reported that 6 of 84 patients had recurrence of bacteremia (*3*). We found recurrence in 9 of the 92 patients. Risk factors were similar to those previously reported for non–group A streptococcal cellulitis (*13*). PFGE of these isolates showed that a high percentage of recurrence was caused by identical strains. Although Cohen-Poradosu et al. reported that *emm* type stG840 was the most common strain (*3*), we found *emm* type stG485 to be most common.

For years in Taiwan, macrolide resistance of streptococci has been a major health problem (*14**,**15*). A previous study found erythromycin resistance in 23.5% of GGS strains (*14*). Although we did not test for erythromycin resistance, we found some resistance even to new macrolides. Since restriction of macrolide use in Taiwan, a linear relationship has been noted between the decline in erythromycin use and the decline in erythromycin resistance in *S. pyogenes* (*15*). Our study, however, found no decline in macrolide resistance from first half of the study period (27.1%) to the second half (37.0%).

In summary, in our study, infection with *S. dysgalactiae* subsp. *equisimilis* was the most common cause of GGS bacteremia. Infection recurred for ≈10%. The mortality rate for patients with GGS bacteremia was relatively low (<10%), but resistance to quinupristin-dalfopristin was extremely high.

## Supplementary Material

Appendix Table In vitro susceptibilities of 92 isolates of group G Streptococcus, April 1998-August 2004, Taiwan
