# Supplementary material for: Bacteremia Caused by Group G Streptococci, Taiwan
Source: Emerg Infect Dis. 2008 May;14(5):837–40. doi: 10.3201/eid1405.070130 (PMC2600252; doi:10.3201/eid1405.070130)
Supplement: Appendix Table — In vitro susceptibilities of 92 isolates of group G Streptococcus, April 1998-August 2004, Taiwan [file 07-0130_appT-s1.pdf]

Appendix Table. In vitro susceptibilities of 92 isolates of group G *Streptococcus,* April 1998–August 2004, Taiwan

| Antimicrobial agent | MIC (μg/mL)* | | | Susceptible isolates, % |
|---|---|---|---|---|
| | Range | $MIC_{90}$ | $MIC_{50}$ | |
| Penicillin | 0.03–0.12 | 0.06 | 0.03 | 100 |
| Cefepime | 0.03–0.5 | 0.06 | 0.03 | 100 |
| Imipenem | 0.03 | 0.03 | 0.03 | 100 |
| Ertapenem | 0.03–0.5 | 0.06 | 0.03 | 100 |
| Azithromycin | 0.03–>128 | 128 | 0.12 | 67.4 |
| Clarithromycin | 0.03–>128 | 4 | 0.06 | 73.9 |
| Telithromycin | 0.03–32 | 0.5 | 0.03 | 96.7† |
| Clindamycin | 0.06–>128 | 64 | 0.25 | 87.0 |
| Ciprofloxacin | 0.06–1 | 0.5 | 1 | 100 |
| Levofloxacin | 0.06–1 | 0.5 | 1 | 100 |
| Moxifloxacin | 0.06–0.5 | 0.25 | 0.12 | 100 |
| Vancomycin | 0.12–1 | 0.5 | 0.25 | 100 |
| Quinupristin-dalfopristin | 0.5–4 | 4 | 2 | 33.7 |
| Linezolid | 1–2 | 2 | 2 | 100 |
| Tigecycline | 0.03–1 | 0.5 | 0.12 | 100‡ |

*$MIC_{50}$, 50% inhibition; $MIC_{90}$, 90% inhibition.
†Defined as MICs <0.5 μg/mL.
‡Defined as MICs <2 μg/mL.
